# The role of monocytes in thrombotic diseases: a review

**DOI:** 10.3389/fcvm.2023.1113827

**Published:** 2023-06-02

**Authors:** Zhongyu Han, Qiong Liu, Hongpeng Li, Meiqi Zhang, Luling You, Yumeng Lin, Ke Wang, Qiaoyin Gou, Zhanzhan Wang, Shuwei Zhou, YiJin Cai, Lan Yuan, Haoran Chen

**Affiliations:** ^1^ School of Medical and Life Sciences, Chengdu University of Traditional Chinese Medicine, Chengdu, China; ^2^ Eye School of Chengdu University of Traditional Chinese Medicine, Chengdu, China; ^3^ Lianyungang Clinical College of Nanjing Medical University, Lianyungang, China; ^4^ Department of Radiology, The First Hospital of Hunan University of Chinese Medicine, Changsha, China; ^5^ Science and Education Department, Chengdu Xinhua Hospital, Chengdu, China

**Keywords:** monocyte, macrophage, thrombotic diseases, arterial thrombosis, intravenous thrombolysis

## Abstract

Cardiovascular and cerebrovascular diseases are the number one killer threatening people's life and health, among which cardiovascular thrombotic events are the most common. As the cause of particularly serious cardiovascular events, thrombosis can trigger fatal crises such as acute coronary syndrome (myocardial infarction and unstable angina), cerebral infarction and so on. Circulating monocytes are an important part of innate immunity. Their main physiological functions are phagocytosis, removal of injured and senescent cells and their debris, and development into macrophages and dendritic cells. At the same time, they also participate in the pathophysiological processes of pro-coagulation and anticoagulation. According to recent studies, monocytes have been found to play a significant role in thrombosis and thrombotic diseases of the immune system. In this manuscript, we review the relationship between monocyte subsets and cardiovascular thrombotic events and analyze the role of monocytes in arterial thrombosis and their involvement in intravenous thrombolysis. Finally, we summarize the mechanism and therapeutic regimen of monocyte and thrombosis in hypertension, antiphospholipid syndrome, atherosclerosis, rheumatic heart disease, lower extremity deep venous thrombosis, and diabetic nephropathy.

## Introduction

Thrombotic diseases, which are mostly caused by thrombosis and thromboembolic obstruction, are the main challenges in clinical practice (1, 2). At present, thrombotic diseases have become a kind of circulatory system disease that seriously endangers human health. Based on research data from the World Health Organization, CVDs are responsible for the highest number of deaths globally, with 17.9 million people dying from these diseases each year. A significant proportion of these deaths result from cardio-cerebrovascular embolism (3). According to the location of the disease, thrombotic diseases can be classified into arterial thrombosis, venous thrombosis and microcirculatory disorders.

For decades, it has been recognized that there is a complex interaction between coagulation and inflammation, and the coagulation cascade and platelet activation can trigger the immune system, leading to leukocyte recruitment, adhesion, extravasation and activation, and the immune system can in turn influence haemostatic system, a process referred to as immunothrombosis (4). Monocytes, which are myeloid cells throughout an individual's lifetime, contribute to regulating immune responses and serve multiple functions in the body, such as tissue development and maintaining vascular homeostasis, host defense, and playing a role in initiating and resolving inflammation and tissue remodeling (5). Monocytes in an immature state are released from the bone marrow into the bloodstream, after which some relocate to various tissues to differentiate into resident macrophages (MΦ) or dendritic cells (DC) (6, 7). Under the action of different factors and environments, macrophages can be polarized into different subtypes, and the common ones are classically activated MΦ1 (M1) or alternately activated MΦ2 (M2). M1 macrophages polarized into a pro-inflammatory state, and exhibited high expression of proinflammatory miRNA, promoting Th1 response (8). On the other hand, M2 macrophages have been found to promote tissue repair and cell proliferation, display anti-inflammatory characteristics, and encourage a Th2 response (8). The influence of the two macrophage subsets on angiogenesis is divergent, with M1 macrophages generally suppressing cell proliferation and reducing the ability of endothelial cells (ECs) to undergo angiogenesis, while M2 macrophages often oppose this effect (8, 9). Studies have demonstrated that M2 macrophages can facilitate angiogenesis by upregulating basic fibroblast growth factor (bFGF), insulin-like growth factor-1 (IGF1), and placental growth factor signaling pathways (10).

Different monocytes subset play diverse roles in cardiovascular physiology and pathophysiology. Though now, there are many subgroups (Table 1) to be researched through various classification methods and the mechanisms are still not enough to understand, several experiments have shown changes in the absolute and relative concentrations of these subsets can serve as exquisite markers of different inflammatory states in CVDs (15–18). In cases of thrombosis, the formation of circulating monocyte-platelet aggregates (MPA) can indicate platelet activation and the presence of an inflammatory response. MPA act as a mediator between inflammation and thrombosis, highlighting their significant role (19). Multiple receptor ligands bind between monocytes and platelets, leading to activation of MPA. P-selectin/PSGL-1 mediated aggregation of platelet and monocytes leads to NF-κB activation/translocation, superoxide anion production and promotion of monocyte chemotactic protein-1 (MCP-1), interleukin-8 (IL-8), IL-1β and tissue factor (TF) release, which trigger coagulation and accelerate thrombosis (20, 21). Moreover, multiple studies have found that heightened levels of MPA in individuals with coronary heart disease, unstable angina pectoris, and acute myocardial infarction contribute significantly to the acceleration of thromboembolic events (22–24). This highlights the importance of MPA in the progression of CVD.

**Table 1:** Phenotype and function of circulating monocyte subsets in human.

Subset	Surface markers	Chemokine receptors	% of total	Functions	Cytokine production	Reference
Classical	CD14^++^CD16^−^	CXCR2^+^CX3CR1^−^	80–95	Phagocytic, tissue repair	IL-1, IL-10, IL-12, TNF-α	(11)
Intermediate	CD14^++^CD16^+^	CCR2^−^CX3CR1^+^	2–11	Highly proinflammatory cells that produce high levels of ROS and inflammatory mediators	TNF-α, IL-1β, IL-6	(12, 13)
Nonclassical	CD14^+^CD16^++^	CCR2^−^CX3CR1^+^	2–8	Patrolling, clearance of debris, cearance of apoptotic cells, anti-viral responses	TNF-α, IL-1β, IL-6	(14)

Neutrophil extracellular traps (NET) are the network ultrastructure released into the extracellular after polymorphonuclear neutrophils (PMN) activation, which serves as the first line of defense against microbial infection in the early stage. Studies have shown that NET is associated with thrombus-inflammation-related diseases including Sepsis, systemic lupus erythematosus, and coronary heart disease, which is a hot topic in current research (25, 26). At present, Monocyte extracellular trap (ET) shows similar morphology to NET, exhibited procoagulant activity, and was associated with myeloperoxidase (MPO), lactoferrin (LF), citrullinated histones, and elastase (27, 28). In an inflammatory setting, extracellular trap cell death (ETosis) occurs when peripheral blood mononuclear cells encounter elastase and citrullinated portions of NET, and monocytes preferentially take up apoptotic bodies from PMN, thereby removing apoptotic bodies and NET-DNA (28, 29). We increasingly recognize the importance of monocytes in participating in thrombotic diseases such as CVDs, and increasing research condenses into monocyte-specific molecular mechanisms, signaling pathways, and gene expression, providing direct evidence to elucidate some diseases (21, 30–33).

Indeed, the role of monocytes in the link between inflammation and the pro-thrombotic state is a topic of interest and relevance in the field of cardiovascular disease. Monocytes are important immune cells that can differentiate into different subsets of macrophages that play distinct roles in the body's immune response. During inflammation, monocytes can become activated and release pro-inflammatory cytokines, which can contribute to the development of a pro-thrombotic state. Additionally, monocytes can interact with platelets and endothelial cells, further contributing to the development of thrombosis. There has been some research on the specific mechanisms by which monocytes contribute to the link between inflammation and the pro-thrombotic state, and further studies in this area could potentially lead to the development of new therapeutic approaches for the prevention and treatment of cardiovascular disease.

It should be mentioned that roles of moncytes in arterial thrombosis “itself” has been rarely investigated. Pathophysiology of thrombotic diseases are complex, because arterial atherosclerosis (or venous blood stasis), plaque (and rupture), tissue injury (such as cerebral infarction and myocardial infarction), and systemic stress (related to cardiovascular events in humans and animals or modeling-related surgical stress in animals) are interwined, affecting each other. Therefore, under the subtitle of monocytes and arterial thrombosis, we do not have much data; monocyte-related features in the literature for “thrombotic diseases” are not necessarily about “thrombosis” itself.

## Monocyte subsets and cardiovascular thrombotic events

Monocytes are the largest blood cells in the blood, accounting for 8%–10% of the total number of leukocytes in the body (34). Monocytes are the main component of the autoimmune response and are closely related to the endogenous inflammatory process, its surface sensing changes of the receptor (35). Monocytes can respond quickly when body tissues are damaged or infected, transforming into macrophages or DC to regulate the inflammatory response and safeguard the body from infection and injury (36). Several studies have shown that monocytes are heterogeneous and plastic, and a variety of molecular markers are present on the cell membrane, such as adhesion molecules, complement receptors, and cytokines (31, 37). These molecules jointly participate in the physiopathological processes such as cell migration, thrombosis, and phagocytosis (31, 38, 39). According to the International Federation of Immunology's 2010 classification, human monocytes were divided into three subsets based on the expression of CD14 (an LPS-related receptor) and CD16 (an FcγⅢ receptor). These subsets include Classical Monocytes (CMs, CD14^++^CD16^−^), Intermediate Monocytes (IMs, CD14^++^CD16^+^), and Non-classical Monocytes (NCMs, CD14^+^CD16^++^) (40) (Table 1). Classical monocytes overexpressed genes related to phagocytosis, such as CD93, CD64, CD11B, CD36, CD32, CD14, ficolin-1 (FCN1), and signal regulatory protein alpha (SIRPA) (41, 42). CMs are involved in various immune responses such as inflammation and tissue repair (43). IMs exhibit the highest expression of TLR2, TLR4, CD40 and MHC-class II molecules (HLA-DR), and also possess the greatest antigen presentation ability (42). NCMs perform endothelial cell patrolling, which aids in maintaining cellular integrity, removing dead ECs, repairing the vasculature in atherosclerotic diseases, and removing lipids from the blood (44). *In vitro* studies have revealed that NCMs demonstrate the most robust response to LPS, and are capable of secreting pro-inflammatory cytokines like tumor necrosis factor-alpha (TNF-α) and IL-1β. The heightened expression of miR-146a observed in NCMs is indicative of the senescence-associated secretory phenotype (SASP) that results from increased basal levels of phosphorylated NF-κB (p65) and IL-1α (45).

The developmental relationship of monocytes goes through three stages, which are typical phenotype, intermediate phenotype and non-classical phenotype. The results of several current studies have shown that researchers have a large controversy about proinflammatory cytokines secreted by monocytes of different phenotypes (42, 46). In a comparison regarding how much reactive oxygen species (ROS) are produced, Cros et al. suggested that IMs do not produce ROS and CMs produce large amounts of ROS, while Zawada et al. suggested that CMs produce the lowest ROS and IMs produce the most ROS (41, 46). In terms of how much TNF-α is secreted, Cros et al. suggested that IMs produce the most TNF-α, whereas Wong et al. claimed that NCMs produce the highest levels of TNF-α (42, 46). In addition, at the level of gene expression subset function, Cormican and Griffin screened genes targeting monocyte subsets while giving a critical evaluation (33). Anika Witten et al. conducted flow cytometric analysis and genome-wide transcriptional profiling to investigate the correlations between miRNA and mRNA species in three distinct monocyte subsets. These subsets were obtained from patients with first acute myocardial infarction (MI), stable coronary artery disease (CAD), and individuals without any previous history of CVD. They found that most MI-specific miRNAs were involved in NCMs, which was consistent with the involvement of NCMs in tissue repair after myocardial infarction. In the inflammatory state of active CAD, the CMs are more inflammation and patrolling. The findings suggest that miRNAs from monocyte subsets might play a significant role in CVD both during and after the disease progression. Due to their stability and abundance in the circulating blood, the miRNAs linked to monocytes could be potentially useful as biomarkers for diagnosis, prevention, and treatment of CVD (47).

In the past few decades, there has been growing evidence pointing to the existence of distinct subpopulations of monocytes that play different roles in the development of CVDs. Changes in the function, number, and proportion of these monocyte subsets have been closely linked to the progress and prognosis of CVD (15, 48–50). A single published literature by Helen Williams very well has reviewed the relationship between the nature and number of monocyte subpopulations developing in CVDs (51). The ratio of IMs and/or NCMs are frequently reported to be elevated in CVD (51). In general, CD16^+^ monocytes may have more promoting inflammatory and pro-coagulant functions in vascular inflammation. In a clinical trial, *IL-6* and *IL-8* mRNA levels were elevated in both IMs and NCMs and not detected in CMs after LPS bolus injection in 12 healthy volunteers (52). In addition, CD16 on CMs can be induced to rise by low serum HDL-C in patients with coronary atherosclerosis, resulting in a concomitant increase in IMs (53, 54). Interestingly, R. Cappellari et al. made an intriguing discovery-they found that cardiovascular outcomes could be predicted by the shift of monocyte subsets along their continuum, as opposed to the proportions of these subsets (55). The shift of monocyte subsets along their continuum in patients with major adverse cardiovascular events (MACE) was presented and described, mainly manifested as the increase of classical CD16 fluorescence intensity, the increase of intermediate type proportion, and the increase of non-typical CD14 fluorescence intensity. Such drift changes are particularly helpful in determining cardiovascular events. The findings indicate that the predictive power of monocyte subsets for adverse cardiovascular outcomes lies in the shift along the CD14/CD16 continuum, rather than their frequency. Ramona Vinci et al. investigated whether variations in plaque erosion and rupture, as identified by optical coherence tomography (OCT) studies, were significantly different in non-ST-segment elevation acute coronary syndromes (ACS) patients based on the analysis of monocyte subsets present in circulating blood (56). The study introduced a pre-classical monocyte (PCM) population near the coronary microvasculature with CD14^+^CD16^−^ surface staining. The distribution of monocyte subsets was analyzed in the study population, and the frequency of the newly identified PCM subsets was found to be higher in patients with non-ST-segment elevation ACS (NSTE-ACS) compared to those with chronic coronary syndrome (CCS). When the plaque phenotype was analyzed by OCT, the PCM subsets were higher in NSTE-ACS patients, especially those with ruptured fibrous cap (RFC) plaques and concomitant local macrophage infiltration (MΦI). Overall, higher rates of circulating PCM may represent a unique marker of specific plaque rupture with local MΦI.

Furthermore, the Monocyte-to-HDL Ratio (MHR), which is a newly emerged biomarker indicating inflammation and oxidative stress, can be conveniently obtained in clinical settings. Currently, it has been considered a marker of drug-resistant hypertension in chronic kidney disease (CKD), coronary heart disease, diabetic retinopathy, pulmonary embolism (PE) and other diseases (57–61).

## Monocytes and arterial thrombosis

Arterial thrombosis is mainly due to the blood vessels in unstable atherosclerotic plaque rupture, plaques inside the lipids are released into the bloodstream, causing blood platelet aggregation and adhesion, and attracting white blood cells (WBC) and red blood cell (RBC) aggregation, leading to thrombosis, vascular occlusion and possible acute myocardial infarction, ischemic stroke, lower limb arteriosclerosis occlusion (62). Arterial thrombosis is characterized by high blood flow velocity and high wall shear rate. When ECs are destroyed or atherosclerotic plaque is ruptured, a series of events lead to the formation of platelet-rich thrombosis (63).

At sites of vascular injury, inflammation, or thrombosis, platelet activation plays a crucial role in orchestrating the recruitment of WBC. This intricate process is largely mediated via the release of soluble mediators or through direct cellular interactions. Notably, platelets exhibit the highest binding affinity with monocytes/macrophages, and their potency descends progressively with respect to neutrophils and lymphocytes. This mechanism of cellular recruitment represents a profound component of the immune response and serves to fortify the host's defenses against pathogenic insults (64).

**Figure 1:**
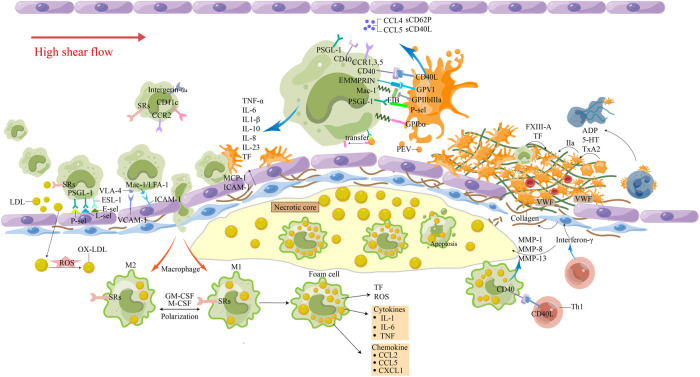
The involvement of monocytes in atherosclerosis-thrombosis. The release of pro-inflammatory factors from cells in the plaque and the damage of ECs caused circulating monocytes to roll, roll slowly, adhere firmly, and travel through the tissue to differentiate. Macrophages phagocytose excessive Ox-LDL and other immune substances, causing apoptosis and necrosis of cells, lipid deposition, the generation of a large number of inflammatory factors and tissue factors, and promoting the transport of more circulating monocytes. Activated Th1 cells produce interferon γ, which inhibits smooth muscle cell synthesis of interstitial collagen. The interaction between Th1 cells and M1 macrophages leads to the overproduction of MMP-1, MMP-8 and MMP-13, and the interstitial collagenase can promote the breakdown of interstitial collagen. At the same time, it can induce M1 macrophages to overexpress tissue factors. These processes make the fibrous cap more vulnerable to rupture, and inflammatory activation leads to the increased potential for thrombosis. Platelet-rich thrombosis forms around atherosclerotic plaque rupture and endothelial damage when endosubcutaneous collagen is exposed to circulation under high shear flow. Platelets, through their glycoproteins, interact with collagen and collagen-deposited vWF to change their shape and adhere to the injured site. Attachment results in the secretion of ADP, serotonin, and thromboxane (TxA2), which recruit and activate more platelets. The platelet-monocyte complex (PMC) is activated by the interaction of various receptor ligands between platelets and monocytes. Platelets and monocytes also shed extracellular vesicles, release cytokines and chemokines, and tissue factors are increased to promote thrombosis. 5-HT, 5-hydroxytryptamine; E-sel, E-selection; FXIII, Factor XIII; FIB, fibrinogen; GM-CSF, granulocyte-macrophage colony stimulating factor; IL-8, interleukin-8; LDL, low-density lipoproteins; L-sel, L-selection; MCP-1, monocyte chemotactic protein-1; oxidized LDL; M-CSF, macrophage colony-stimulating factor; MMP, matrix metalloproteinases; Ox-LDL, oxidized LDL; PEV, platelet-derived extracellular vesicles; PMC, platelet-monocyte complex; P-sel, P-selection; ROS, reactive oxygen species; SRs, scavenger receptors; TF, tissue factor; TNF-α, tumor necrosis factor-α; TxA2, thromboxane; VCAM-1, vascular cell adhesion molecule-1; vWF, von Willebrand factor.

P-selectin (CD62P), a transmembrane glycoprotein of platelets, constitutes a fundamental component of thromboinflammatory cascades. Its exposure on the surface of platelets initiates a series of intricate interactions with leukocytes by binding to O-glycosylated carbohydrate ligands on PSGL1 on the outer surface of myeloid cell membranes. Such binding, synergistic with the CD40-CD40l pathway, elicits intracellular signal transduction, culminating in the upregulation of TF expression in leukocytes (20, 30, 65–67). In turn, PSGL-1 induce up-regulation and activation of integrin β2 [macrophage-1 antigen (Mac-1) and lymphocyte function-associated antigen-1 (LFA-1)] in monocytes, further supporting their interaction with platelets (68). Glycoprotein (GP) VI, a prominent receptor located on the surface of platelets, drives the interaction between platelets and the extracellular matrix metalloproteinase inducer (EMMPRIN, CD147/basigin) expressed on the surface of monocytes, promoting their recruitment to the site of inflammation in arterial walls (69, 70). In addition to the direct interaction, platelet-derived substance influence monocyte recruitment and endothelial cell adhesion and behavior. Intriguingly, platelets activated under conditions of vascular insult then extrude into their surroundings, an array of extracellular vesicles (EVs) derived from the membrane-bound structures of the activated platelets. These EVs exhibit a preference towards binding to blood monocytes in comparison to other leukocyte subsets, a mechanism chiefly contributing to the selective recruitment of monocytes towards sites of inflammation or tissue injury. This intricate process represents an indispensable component of the host's immune response, highlighting the functional importance of these EVs as modulators of both inflammation and thrombosis (Figure 1). Platelet-derived EVs transfer the platelet adhesion receptor GPIbα to the surface of monocytes, which can be recruited in various blood vessels (71). As a perfectly tuned transcellular conveyance mechanism for RANTES, circulating platelet microparticles (PMPs) augment the adhesion of CXCL4 (PF4) to the exterior of monocytes, inducing a robust anti-inflammatory effect. The exquisite intercellular orchestration orchestrated by PMPs not only furthers the targeted transport of RANTES, but also orchestrates an intricate cellular dialogue that culminates in the suppression of the inflammatory signals generated by activated monocytes (72, 73). CXCL4 induces monocyte respiratory burst through rapid activation of phosphoinositide 3-kinase (PI3K), spleen tyrosine kinase (SYK), and p38 mitogen-activated protein kinase (MAPK), mediates ROS formation, cytokine/chemokine expression, and apoptosis rescue depends on continuous activation of SPHK and extracellular regulated protein kinases (ERK) to inhibit caspase activation (74, 75). CXCL4 also promotes monocyte differentiation into macrophages and inhibits CD163 in macrophages to promote atherogenesis (76, 77). Through a precision-driven process, activated platelets demonstrate their nuanced ability to manipulate the MCP-1 and intercellular adhesion molecule-1 (ICAM-1) properties of ECs using a mechanism that is dependent upon the regulation of NF-κB (78). With intricate precision, downstream upregulation of adhesion molecule target genes, namely vascular cell adhesion molecule-1 (VCAM-1), ICAM-1, E-selectin, P-selectin and MCP-1, confers an indirect yet potent effect on monocyte recruitment (79). Overall, the formation of MPA is sophisticated, but it is crucial for thrombosis. With remarkable dexterity, previous studies have postulated that P-selectin may be a key determinant in the initiation of the intricate cellular conjugation between platelets and monocytes, subsequently fueling a deleterious cycle of platelet activation that manifests as a cascade of platelet activator synthesis. By offering an incisive glimpse into this complex interplay, these seminal findings expand our understanding of the intricate molecular pathways that underlie the profound intercellular cooperation observed in platelet-monocyte conjugation. Such knowledge opens up a rich avenue of exploration for the development of novel therapies designed to target the various components involved in this process for better clinical outcomes.

With intricate precision, it has been postulated that leukocytes navigate across the luminal surface of inflammatory ECs via a refined rolling mechanism, only to then stabilize their interaction with the endothelium and progress into the vascular wall. This decisive cellular behavior is regulated by a fine-tuned process of cellular crawling, through which the leukocytes reach the appropriate extravasation site with remarkable specificity. Crucial to this intricate interplay is the dynamic regulation of adhesion molecules, which confer nuanced yet prodigious control over the molecular pathways underlying leukocyte motility and extravasation (31).

With unparalleled finesse, it has been theorized that the exquisite interplay between inflammation and ECs lays the foundations for a highly orchestrated cascade of molecular events. Of primordial import is the rapid induction of the expression of key endothelial adhesion molecules, which are fine-tuned with remarkable precision by the pivotal inflammatory factors, TNF-α and IL-1β. In a study, the authors have expounded on an intricate regulatory scheme governing the rolling rate of inflammatory monocytes. Specifically, their results demonstrate that the resynthesis of E-selectin by ECs plays an indispensable role in activating said regulatory mechanism. Additionally, they have delineated a multifaceted process by which P-selectin, L-selectin, PSGL1, and CD44, intricately intertwine and combine forces to regulate the flux of rolling neutrophils and inflammatory monocytes (80, 81). Very late antigen-4 (LA-4 α4β1 integrin) expressed by monocytes can bind to VCAM-1 on the endothelium, mediating its slow roll to firm adhesion on ECs activated by cytokines such as CXCL1 (82, 83). Monocytes are able to roll over the endothelium and appropriately adapt to the high shear stress that is imposed by blood flow, due to their interactions with C-C and C-X-C chemokines such as C-C chemokine ligand 2 (CCL2) (MCP-1) and CXCL8 (IL-8). These chemokines facilitate the tight adhesion of monocytes to ECs (84, 85). ICAM-1 also promotes leukocyte adhesion and migration through its ligands (LFA-1, MAC-1) and participates in the interaction between ECs and monocytes (86, 87). The risk factor C-reactive protein (CRP) leads to a reduction in endothelial nitric oxide production and drives plaque instability through several mechanisms. These mechanisms include increasing the expression of endothelial cell adhesion molecules, inducing monocyte TF through CRP, promoting the recruitment of monocytes to atherosclerotic plaques, and enzymatically binding modified low-density lipoprotein (88).

TF-positive monocyte-derived microvesicles (MVs) are small, membrane-bound fragments that are released from monocytes into the bloodstream. Monocytes are a type of white blood cell that can differentiate into macrophages, which play a key role in the immune response to infection and inflammation. When monocytes encounter oxidized LDL cholesterol particles in the bloodstream, they can become activated and release TF-positive MVs. These MVs can circulate widely in the bloodstream and contribute to coagulation-related concerns. TF-positive MVs are one of the sources of TF in the bloodstream, along with monocytes and other cells. The upregulation of tissue factor expression in circulating monocytes and macrophages through the TLR4/TLR6/CD36 receptor complex that occurs in the presence of OxLDL is thought to play a key role in the development of atherosclerosis and other vascular inflammatory diseases. As a result, TF-positive MVs are an important biomarker of vascular inflammation and thrombotic risk (89). We summarize the latest factors that stimulate monocytes to produce TF coagulation (90) (Table 2). Plaque rupture exposes the blood to high levels of TF-induced thrombin, initiating a coagulation cascade that subsequently forms a fibrin monolayer covering the exposed damaged surface of the vessel (130) (Figure 1).

**Table 2:** Effect of different factors and milieu on monocyte tissue factor expression and procoagulant activity.

Factors/milieu	Model	*In vitro*/*vivo*	Mechanism/result	Effect (E&A)	Reference
IL-6/IL-8	Human	*In vitro*	Induce monocyte PCA by increasing mRNA, protein content, and surface expression of TF	Increased E	(91)
IL-1/TNF-α	Human	*In vitro*	Induces TF activity in human monocytes	Increased A&E	(92)
IL-4/IL-13	Human	*In vitro*	Effectively diminished IL-1 alpha/beta induced PCA, shown at the protein and at the mRNA-level	Reduced A&E	(92)
IL-33	Human	*In vitro*	IL-33 induced a time-and concentration-dependent increase of monocyte TF mRNA and protein levels via binding to the ST2-receptor and activation of the NF-κB-pathway	Increased E	(93)
MCP-1	Human	*In vitro*	MCP-1 induces the accumulation of TF mRNA and protein in THP-1 monocytic leukemia cells	Increased A&E	(94)
PDGF-BB	Human	*In vitro*	–	Increased E	(95)
PDGF-CC	Human	*In vitro*	PDGF-CC induces TF expression via activation of α/β receptor heterodimers and an ERK-dependent signal transduction pathway	Increased A&E	(96)
IL-10	Human	*In vitro*	Endogenous IL-10 regulates TF expression and release of active TF-bound microparticles by a negative feed back loop through IL-10 receptor alpha	Reduced A&E	(97)
P-selectin	Human	*In vitro*	P-selectin on activated platelets rapidly triggers TF exposure on monocytes independent of *de novo* protein synthesis	Increased E	(98)
aPL	Human	*In vitro*	Dependent on inducing tumor necrosis factor-α (TNF-α) secretion	Increased E	(99)
	Human	*In vitro*	TLR4 signal transduction pathways participate in anti-β2GPI/β 2GPI-stimulated TF and TNF-α expression in monocytes, and both MyD88 and TRIF adaptors are involved in the process.	Increased E	(100)
	Human	*In vitro*	aPL induces TF expression in monocytes from APS patients by activating, simultaneously and independently, the phosphorylation of MEK-1/ERK proteins, and the p38 MAP kinase-dependent nuclear translocation and activation of NF-kappaB/Rel proteins	Increased E	(101)
Anti-DNA antibodies cross-reactive with β2-glycoproteinI	Human	*In vitro*	TLR9 activation by DNA which was internalized together with cross-reactive antibodies produced in secondary APS accompanying SLE	Increased E	(102)
LPS	Human	*In vitro*	Dependence on LPS-Binding protein and CD14, and Inhibition by a recombinant fragment of bactericidal/permeability-increasing rotein	Increased A&E	(103)
ATG	Human	*In vitro*	ATG induces monocyte TF procoagulant activity dependent on complement activation but independent of *de novo* protein synthesis	Increased	(104)
MPA					
PCSK9	Human	*In vitro*	PCSK9 induces TF expression through activation of TLR4/NFkB signaling	Increased A&E	(105)
Homocysteine	Human	*In vitro*	Homocysteine induce TF expression by human peripheral blood monocytes in a specific manner at physiologically relevant concentrations	Increased E	(106)
Neutrophil elastase	Human	*In vitro*	Neutrophil elastase mainly enhances tissue factor production by monocytes via the phospholipase C/conventional PKC/p38 MAPK pathway	Increased E	(107)
Amiodarone	Human	*In vitro*	Amiodarone inhibits tissue factor expression in monocytic cells, by inhibiting mRNA transcription	Reduced E	(108)
SP	Human	*In vitro*	SP binding to neurokinin-1 receptor induces monocytes to release cytokine/chemokine mediated TF expression	Increased A&E	(109)
15(S)-HETE	Human	*In vitro*	15(S)-HETE–induced TF expression and its activity require reactive oxygen species– dependent calcium/calmodulin-dependent protein kinase IV (CaMKIV)–mediated nuclear factor of activated T cells 3 (NFATc3) and FosB interactions and their occupancy of AP-1 site in the TF promoter	Increased A&E	(110)
aODN	Human	*In vitro*	TF mRNA antisense ODN specifically suppressed the synthesis of biologically active monocyte TF	Reduced E	(111)
Simvastatin	Human	*In vitro*	Inhibit the monocyte expression of TF by interfering with intracellular synthesis of cholesterol	Reduced E	(112)
hsa-miR-223-3p	Human	*In vitro*	hsa-miR-223-3p can bind to a complementary site within the 3′-UTR of the TF mRNA transcript to control its expression	Reduced	(113)
HNE	Human	*In vitro*	NE increases TF coagulant activity in monocytic cells through a novel mechanism involving p38 MAPK activation that leads to enhanced PS exposure at the cell surface without increasing TF protein levels	Increased A	(114)
bFGF	Rabbit	*In vivo*	–	Increased A&E	(115)
VIP/PACAP	Human	*In vitro*/*in vivo*	VIP and PACAP inhibit LPS-induced TF expression in monocytes *in vitro* and *in vivo*, block both the migration of c-Rel/p65 and the phosphorylation of p38 and JNK	Increased A&E	(116)
Histone	Human	*In vitro*	Histones, particularly subunits H3/H4, increases surface TF activity via increased surface TF antigen and PS exposure.	Increased A&E	(117)
AGEs	Human	*In vitro*	AGE-induced TF expression in monocytes is mediated by oxidant stress	Increased	(118)
GGT	Human	*In vitro*	Human recombinant GGT induced TF expression in monocytes through a cytokine-like mechanism that involved the activation of TLR4/NF-κB signaling	Increased A&E	(119)
Insulin	Human	*In vitro*	Insulin inhibits TF expression in monocytes and monocyte-derived microparticles through interference with Giα2-mediated cAMP suppression, which attenuates Ca2+-mediated TF synthesis	Reduced	(120)
Bradykinin	Mice	*In vivo*	PI3K/Akt signaling pathway activation induced by bradykinin administration reduced the activity of GSK-3β and MAPK, and reduced NF-kB level in the nucleus, thereby inhibiting TF expression; Bradykinin inhibited tissue factor expression of monocytes by BK B2 receptor–mediated NO release	Reduced A&E	(121)
CS	Human	*In vitro*	BDNF released by platelets upon activation by CS modulates TF activity in human peripheral blood monocytes (PBMs)	Increased A&E	(122)
BMP-7	Human	*In vitro*	BMP-7-mediated increase in TF mRNA/protein levels and functional activity in circulating human monocytes is due to the activation of NF-kB, but not AP-1	Increased A&E	(123)
ATRA	Human	*In vitro*	ATRA downregulated monocyte TF expression and reduced thrombus formation on adherent monocytes at arterial shear	Reduced A&E	(124)
PF4/heparin-antibody complex	Human	*In vitro*	Induction of TF is mediated via engagement of the FcγRI receptor and activation of the MEK1-ERK1/2 signaling pathwa	Increased E	(125)
Local anesthetics (lidocaine, ropivacaine, and bupivacaine)	Human	*In vitro*	Reduced the expression of TF antigen and activity in activated monocytes by inhibiting TF mRNA synthesis	Reduced A&E	(126)
Adipokine apelin-13	Human	*In vitro*	Apelin-induced TF expression was mediated by the G-protein-transcription factor NF-κB axis	Increased A&E	(127)
THC	Human	*In vitro*	THC mediated elevation of TF expression at a post-transcriptional level by inducing stabilization or preventing degradation of TF mRNA	Increased A&E	(128)
sGC (BAY 41-2272 and BAY 58-2667)	Human	*In vitro*	The downregulation of TF expression and functional activity by BAY 41-2272 and BAY 58-2667 are mediated through the sGC-dependent mechanisms involving the suppression of transcriptional activityof NF-κB.	Reduced A&E	(129)

3′-UTR, 3′-untranslated region; β2GPI, phospholipid-bound β2-glycoprotein I; 15(S)-HETE, 15(S)-hydroxyeicosatetraenoic acid; AGEs, advanced glycosylation end products; aODN, antisense oligodeoxynucleotide; AP-1, activation protein-1; ATG, antithymocyte globulin; aPL, antiphospholipid antibody; BDNF, brain-derived neurotrophic factor; bFGF, basic fibroblast growth factor; BMP-7, bone morphogenetic protein-7; CS, cigarette smoke; CaMKIV, calcium/calmodulin-dependent protein kinase IV; ERK, extracellular signal-regulated kinase; HNE, 4-hydroxy-2-nonenal; MAPK, mitogen-activated protein kinase; PACAP, pituitary adenylate cyclase-activating polypeptide; PBMs, human peripheral blood monocytes; PCA, procoagulant activity; PDGF-BB, platelet-derived growth factor BB; PDGF-CC, platelet-derived growth factor CC; PF4, platelet factor 4; PKC, protein kinase C; PS, phosphatidylserine; sGC, soluble guanylate cyclase; SLE, systemic lupus erythematosus; SP, neuropeptidesubstance P; TLR4, toll-like receptor 4; VIP, vasoactive intestinal peptide.

In the early stages of atherosclerosis, monocytes are recruited to the site of arterial injury, where they differentiate into macrophages, which engulf LDL, the “bad” cholesterol that contributes to the development of plaques. As the plaques grow larger, they eventually rupture or become unstable, leading to the formation of arterial thrombosis.

Recent research has suggested that monocyte-derived platelets, which are produced by the transformation of monocytes into megakaryocytes, may also play a key role in the development and progression of arterial thrombosis (131). These platelets are smaller than normal platelets and are more reactive, making them more likely to form blood clots.

The relationship between monocytes and arterial thrombosis has been studied extensively, with several mechanisms that underlie the association between these two phenomena being proposed. One of the mechanisms involves the production of various interleukins, such as interleukin 1β (IL-1β), by monocytes, which can induce endothelial cell damage and contribute to the development of arterial thrombosis. Another mechanism is the involvement of monocytes in the activation of the coagulation cascade, leading to the formation of blood clots.

Monocyte-targeted therapies have been proposed as potential treatments for arterial thrombosis, including the use of inhibitors that target interleukins or suppress the activation of the coagulation cascade. In addition, lifestyle modifications such as regular exercise, a healthy diet, and smoking cessation are recommended to reduce the risk of developing arterial thrombosis.

In conclusion, the relationship between monocytes and arterial thrombosis is a complex phenomenon that involves multiple aspects, and more research is needed to fully understand the mechanisms and potential therapies for this condition. Nonetheless, the increasing evidence of the significant contribution of monocytes to arterial thrombosis highlights the importance of further investigating their role in this disease. Understanding the relationship between monocytes and arterial thrombosis may ultimately lead to the development of more effective treatments and preventative strategies for this condition, which is a significant cause of morbidity and mortality worldwide.

## Monocytes and venous thrombosis

**Figure 2:**
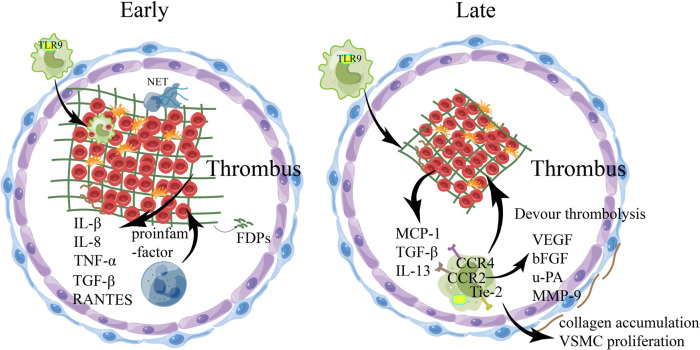
The involvement of relevant immune cells during venous thrombosis. During venous thrombus regression, numerous pro-inflammatory factors are released into the local environment, with an early influx of Neutrophils and macrophages, followed by monocytes, which regulate the production and activity of plasminase and MMPs. In the early stage of thrombolysis, fibrinolysis occurs at a high rate under the action of neutrophils, resulting in the production of fibrin degradation products, the emergence of collagen fibrils within the thrombolus, and the induction of inflammatory cytokines and various proteases by thrombolt-associated immune cells. With the structuring of the thrombus, the dissolution rate of fibrin slows down, collagen proliferation in the thrombus increases, monocytes infiltrate phagocytosis and mobilize EPC, and matrix remodeling of mmp secreted by macrophages occurs, which may eventually recover through the blood flow of the thrombus. bFGF, basic fibroblast growth factor; EPC, endothelial progenitor cells; FDPs, fibrin/fibrinogen degradation products;IL-8, interleukin-8; MCP-1, monocyte chemotactic protein-1; MMP, matrix metalloproteinases; NET, Neutrophil extracellular traps; TNF-α, tumor necrosis factor-α; TGF-β, transforming growth factor-β; TLR-9, toll-like receptor 9;uPA, uridylyl phosphate adenosine; VEGF, vascular endothelial growth factor; VSMC, Vascular smooth muscle cell.

The development of venous thrombosis is primarily linked to reduced shear flow, typically occurring around intact endothelial walls. Venous thrombi often contain high levels of fibrin, and are surrounded by numerous red blood cells and activated platelets. When blood flow decreases, it can result in hypoxia and lead to an increase in the expression of endothelial adhesion molecules. This increased expression can cause leukocytes to attach to the endothelium (62). The coagulation cascade is initiated by the release of TF from leukocyte microparticles and endothelial-binding monocytes. At the same time, thrombin is activated and fibrinogen is converted to fibrin (132) (Figure 2). Hypoxia can lead to the death of ECs and neutrophils, and the dead cell debris needs to be removed by macrophages (133). Pro-inflammatory Mo/MΦ sampled from blood showed significantly elevated M1-polarization in pathological deep vein thrombosis (DVT) in human patients (134). In both animal and human experimental observations concerning thrombus lysis, the presence of macrophages at the site was detected. Notably, the subsequent inflammatory signals triggered by these macrophages led to a heightened formation of thrombus, thereby instigating the ultimate stage of thrombus resolution (134–138). Faster thrombus resolution is the key to improving the prognosis of the disease. The phenomenon of venous thrombolysis and recanalization parallels the intricate biological process of wound healing, whereby the inflammatory response of the venous wall is provoked, establishing the infiltration of diverse inflammatory cells, fibril growth factors, collagen deposition, and the discernible expression and activation of matrix metalloproteinases (MMP) (139–142). Despite the comparatively diminished platelet count in the manifestly proliferating venous thrombosis than in its arterial counterpart, its activated platelets also duly evoke the expression of P-selection, thereby promoting peripheral leukocyte invasion into the thrombus (143).

The phenomena of thrombus dissolution release myriad proinflammatory factors into the indigenous milieu, encompassing IL-1β, TNF-α, JE/MCP-1, IL-6, MIP-1α, and ENA-78 (144). Then, leukocytes begin to invade the thrombus, and PMN is the first to reach the thrombus site, promoting fibrinolysis and collagen dissolution, which are critical for early thrombus dissolution (145). In addition, in the context of PMN inflammation in venous thrombosis, NETs can mediate thrombus fibrosis through TGF-β, and then transform into insoluble chronic thrombosis (146). PMN also promotes the entry of monocytes into the thrombu. Wakefield et al. noted that polymorphonuclear leukocytes in the thrombus began to switch from neutrophils to monocytes on day 4 of thrombosis and reached a peak on day 8 (147). As more macrophages accumulate within the thrombus, they become the primary inflammatory cells and start generating a variety of substances such as chemokines, inflammatory cytokines, and matrix-degrading proteases. Examples of these proteases include uridylyl phosphate adenosine (uPA) and MMPs, which promote both fibrinolysis and tissue remodeling. Additionally, the macrophages release multiple vascular-forming factors such as IL-1, TNF, vascular endothelial growth factor (VEGF), FGF-2, and PDGF. These factors can directly or indirectly stimulate endothelial cell proliferation, migration, and promote the formation of tubule-like structures, eventually resulting in the restoration of blood flow within the thrombosis vein (148–151). MMP-9 causes loss of venous wall compliance by increasing collagen elastin fibers and extracellular matrix (ECM), and subsides thrombosis by reducing macrophage and collagen content (152).

During the later stages of venous thromboembolism (VTE), certain cytokines, including IL-10, TNF-α, IL-6, and IL-8, seem to facilitate the resolution of the thrombus (153–156). In an animal experiment, investigators have found that IL-6 may be a therapeutic target to prevent fibrotic complications that arise in post-thrombotic syndrome (PTS) (157). Besides, Mizuho Nosaka's experiment demonstrated that administering an anti-IL-6 antibody slowed down the resolution of the thrombus (156). This evidence suggests that sophisticated cytokine regulation of thrombus also plays a role in post-thrombotic resolution and ensuing complications.

Attracted by high levels of MCP-1 and other factors (such as local inflammatory reactions), monocytes greatly infiltrate the thrombus and promote thrombus recanalization, and venous flow recanalization is mainly dependent on intrathrombotic neovascularization to establish functional flow channels. It is thought that the resolution of inflammation and acceleration of this process restores venous wall patency and reduces the pathology associated with PTS. According to previous research, while exogenous MCP-1 may speed up the resolution of DVT, it has been found to promote fibrosis in organs *in vivo* (147). In contrast, inhibiting P-selectin and E-selectin has been shown to decrease thrombosis and vein wall fibrosis (158). As such, targeting P-selectin and E-selectin may be promising areas for future research.

## Monocyte and thrombotic diseases

### Vascular hypertension

Generally speaking, when the normal blood pressure is <120/80 mmHg, the blood passes normally. When the blood pressure increases, it will impact the blood vessels and cause pressure on the blood vessels (159). The long-term effect causes damage to the intima of blood vessels and the formation of red thrombosis. Damaged areas form scarring hyperplasia, which thickens the blood vessel wall and continuously forms proliferation and accumulation, narrowing the lumen and increasing the risk of thrombosis (160).

Increased immune cell infiltration may affect some mechanisms directly associated with the development of hypertension, such as promoting the release of aldosterone, increasing the reabsorption of sodium and water, and increasing circulating blood volume. T cells and macrophages produce a variety of pro-inflammatory factors when stimulated, which can lead to cardiomyocyte fibrosis, vascular dysfunction and end-organ damage. Monocytes and macrophages are particularly involved in antigen presentation to T cells and cytokine production in hypertension. Proteins modified by oxidation of highly reactive γ-ketoaldehydes accumulate in DC in multiple hypertensive mouse models. Monocyte-derived dendritic cells (MDCS) make a difference in promoting hypertension and end-organ damage by producing abundant cytokines, promoting the proliferation of CD8^+^ T cells releasing IFN-γ and IL-17A (161).

Macrophages are derived from monocytes, and in the context of vascular inflammation, monocytes are recruited to inflamed tissue and differentiate into macrophages. These macrophages then secrete pro-inflammatory mediators (IL-1), promoting vascular inflammation (162). In a review published by Philip Wenzel et al., monocytes as immune targets in the occurrence, development, and manifestation of hypertension may represent a potentially novel therapeutic avenue for treating hypertension, thereby alleviating hypertension-related end-organ damage or preventing the development or deterioration of hypertension (163). Induction of the diphtheria toxin receptor in Lysm-positive macrophages followed by low doses of diphtheria toxin-depleted bone marrow mononuclear cells reduced circulating mononuclear cells and limited ATII-induced macrophage invasion of the vascular wall. Wild-type CD11b^+^Gr-1^+^ mononuclear cells transfected into depleted LysM^iDTR^ mice restored Angiotensin II (Ang II)-induced vascular dysfunction and arterial hypertension (164). IL-1 is a typical mononuclear/macrophage cytokine, and activation of IL-1 receptors has been implicated in the promotion of sodium retention and elevation of blood pressure through the release of the NKKC2 sodium cotransporter (165).

Mononuclear cell infiltration, invasion, and differentiation in the heart and blood vessels are associated with the progression and severity of hypertensive diseases. In cell co-culture models, activated platelets from patients with essential hypertension are capable of releasing MCP-1, which is responsible for recruiting monocytes to sites of inflammation in blood vessels, thereby mediating the progression of atherosclerosis (166). Monocyte infiltration into the heart and blood vessels during hypertension is dependent on the CCL2-CCR2 axis, and it has been demonstrated in various studies that manipulating drugs or genes can have positive impacts on the chemokine and chemokine receptor pathway (162, 167, 168). In various rat models of hypertension induced by high-salt diet, deoxycorticosterone acetate, and human renin models, monocyte infiltration and adhesion can lead to increased blood pressure and renal injury (169–171).

In human cohort studies, the expression level of cysteine-rich intestinal protein 1 (CRIP1) in monocytes was associated with an increase in blood pressure, and this association decreased with the expression level of CRIP1 when monocytes differentiated into macrophages (172). This suggests that CRIP1 may affect the interaction between monocytes and the pathogenesis of hypertension through proinflammatory regulation and upregulation. In hypertensive patients, increased vascular stretching promotes endothelial cell activation, which enhances the conversion of monocytes into IMs and CD209-labeled monocytes, it activates STAT3 in monocytes and significantly stimulates the expression of IL-1β, IL-23, CCL4 and TNF-α in monocytes to produce these pro-hypertensive cytokines (173). In patients with poorly controlled hypertension and target organ damage (TOD) characteristics, the surface expression of NCMs costimulatory marker CD86 is higher, and the proportion of VEGF-R2 positive non-classical cells is higher, indicating that phenotypic changes in monocyte subsets are related to the progression and severity of hypertension (168).

Summarizing, in the current clinical trials and animal models related to hypertension that we mentioned, we found that organ damage resulting from hypertensive events is closely related to various immune cells of the immune system. Cells of the innate and adaptive immune systems enter target organs in the body through various routes (such as kidneys, blood vessels, myocardium, etc.) and damage target organs by releasing cytokines, matrix metalloproteinases, and ROS, ultimately leading to increased blood pressure. Several factors can activate immune cells to enter target organs, such as a high sodium diet causing an increase in sodium ions in the immune microenvironment. In addition, ECs can release both ROS and IL-6. These molecules can stimulate monocytes to become APCs, which in turn produce cytokines like IL-1β and IL-23. These cytokines can have a significant impact on T cell function. Therefore, reducing the secretion and production of cytokines by such cells in various ways contributes to improving hypertension and end-organ damage.

### Antiphospholipid syndrome (APS)

APS is a rare and complex systemic disease characterized by the presence of antiphospholipid (aPL) antibodies [including lupus anticoagulant, anticardiolipin antibodies, and anti-β2 glycoprotein i (anti-β2GPI) antibodies], with the main clinical features of thrombosis or pathological pregnancy (174). After antibodies bind to β2GPI protein on monocytes and ECs, the Toll-like receptor 4 (TLR-4) triggers the myeloid differentiation primary response protein 88 (MyD88) signaling pathway which leads to the activation of several protein molecules including p38 MAPK, MEK-1/ERK, and NF-κB (174–178). ECs produce microparticles (MP) and MCP-1, while monocyte adhesion to ECs increases due to the activation of the Toll-like receptor (TLR) expression. Furthermore, the presence of proinflammatory cytokines (IL-1β, IL-6, IL-8, and anti-TNF) and chemokines leads to the release of adhesion molecules including E-selectin, VCAM-1, ICAM-1, and TFs. These factors contribute to the inflammation of the blood vessels and promote the growth of ECs, which can ultimately lead to intimal hyperplasia (176, 179, 180). A study of how WB-6 (a mouse monoclonal anti-β2Gpi antibody) contacts and activates resting-state monocytes showed that monocytes internalizing WB-6 express TF and TNF-α, TNF-α stimulates ECs to express ICAM-1 and VCAM-1 (181). Later, another study identified the signaling pathways within cells involved in this process and suggested that the induction of TF expression was the result of the internalization of DNA-activated TLR9 together with cross-reactive antibodies produced by SLE secondary APS (182). It has proved that the β2GPI dimerization domain V(β2GPI-dV) dimerization of β2GPI-dV is sufficient to induce the up-regulation of procoagulant activity of monocytes (183). Antiphospholipid antibodies can activate monocytes and macrophages through systemic allergic reaction toxins (C3a, C4a) produced during complement activation (184).

Chary Lopez-Pedrera et al. first used proteomic analysis to identify changes in the proteomic pattern of monocytes directly associated with thrombotic events in APS and served as a basis for the mechanism of thrombosis in APS (185). Vera M. Ripoll performed a comprehensive proteomic analysis of human monocytes from APS patients with different manifestations using two-dimensional differential gel electrophoresis (2D DiGE). The regulation of four of these proteins showed significant differences in monocytes treated with thrombotic or obstetric APS IgG compared to those treated with healthy control IgG. The proteome of monocytes treated with thrombotic APS IgG was further described using label-free proteomics. Among the 12 most reliable proteins associated with the cytoskeleton, immune response, and coagulation function, POTEE and b-actin-like protein 3 overlapped with 2D DiGE, Abnormalities in the regulation of these proinflammatory and procoagulant proteins could serve as targets for subsequent treatment and contribute to a deeper understanding of the diversity of APS pathogenesis (186). Urine proteomics has recently been reported as a noninvasive method to distinguish primary thrombotic APS from primary obstetric APS. Urinary CXCL12 and PDGF may be potential noninvasive markers to distinguish between them, but the number of patients is small and may be biased (187).

APL can combine with β2GPI and oxLDL to produce oxLDL/β2GPI/anti-β2GPI complex, which can inhibit autophagy and increase the release of inflammatory factors in ECs through a variety of signaling pathways (such as PI3K/AKT/mTOR and eNOS signaling pathways). When a large number of inflammatory factors accumulate around ECs, monocyte-macrophages activate to release TFs, chemokines, growth factors, and metalloproteinases, and differentiate into foam cells, exacerbating atherothrombosis (177, 188, 189).

**Figure 3:**
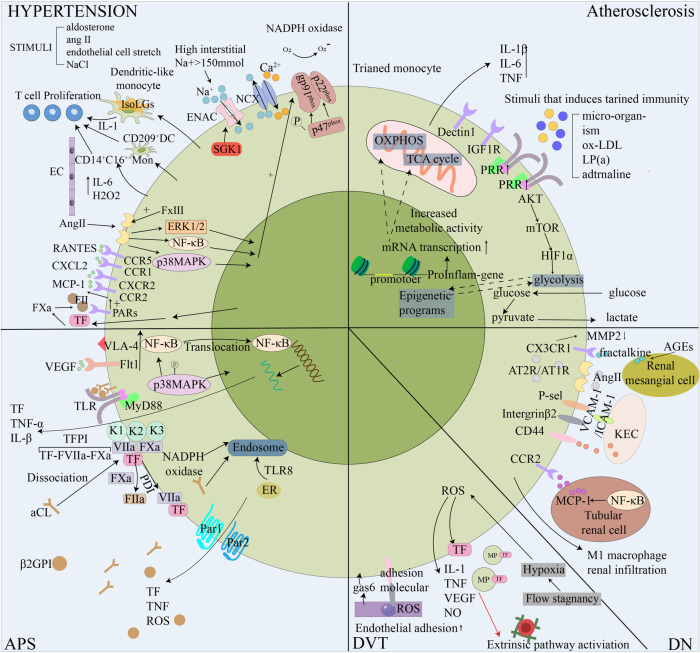
Schematic diagram of the role of monocytes in five thrombotic diseases. In response to the hypertensive stimulant, monocytes transform and activate, release hypertensive cytokines and differentiate into dendritic cells to promote T cell proliferation through the effects of the vascular system, kidney and sympathetic nervous system, elevateing blood pressure, thus increasing the risk of blood vessel rupture. Monocytes are involved in atherosclerosis by training immune processes. This is mediated by epigenetic and metabolic reprogramming. By enhancing cell-related endometabolic pathways, monocytes can develop a long-lasting proinflammatory phenotype. In APS, the anti-β2gpi antibody binds to phospholipid-binding protein β2GPI and acts on monocytes to trigger the Toll-like receptor 4-myeloid differentiation primary response 88 (TLR-4-MyD88) signaling pathway. In addition, aPL triggers coagulation and inflammatory signals by dissociating suppressed TF cell surface complexes. TFPI expressed in myeloid cells specifically supports aPL-induced thrombosis. The hypoxic environment of DVT stimulates the expression of TF and some pro-inflammatory cytokines in monocytes, initiates the exogenous coagulation pathway, activates ECs and promotes the recruitment of monocytes. In the diabetic environment, the accumulation of advanced glycation end products (AGEs) increases the expression of fractalkine in human renal mesangial cells, and the interaction between monocytes and human renal mesangial cells promotes DN inflammation through MMP2 and fractalkine. Ang-II is over-produced and stimulated in the diabetic kidney, the adhesion of monocytes to ECs is enhanced, and M1 macrophages infiltrate the kidney under the regulation of the RAS system. AGEs, Advanced glycation end products; Ang II, Angiotensin II; aPL, antiphospholipid antibody; APS, Antiphospholipid syndrome; AT1R, angiotensin type 1 receptor; β2GPI, beta2glycoprotein I; DC, dendritic cells; DN, Diabetic nephropathy; DVT, Deep vein thrombosis; ENAC, epithelial sodium channel; ER, Endoplasmic reticulum; gas6, growth arrest specific 6; KEC, Kidney endothelial cell; LP(a), Lipoprotein a; IGF1R, insulin like growth factor 1 receptor;MCP-1, monocyte chemotactic protein-1; MMP, matrix metalloproteinases; MP, microparticles; NCX, Sodium-calcium exchangers; NF-κB, nuclear factor κB; NOX-2, NADPH oxidase-2; OXPHOS, oxidative phosphorylation; PARs, protease activated receptors; PRR, pattern recognition receptor; RAS, renin-angiotensin system; ROS, reactive oxygen species; TCA, tricarboxylic acid; TF, tissue factor; TFPI, TF pathway inhibitor; TLR, toll-like receptors; VEGF, vascular endothelial growth facto;VLA-4,very late antigen 4.

At present, it is difficult to diagnose and treat APS patients in clinical practice. Molecular analysis of monocytes in APS patients is helpful to identify unique clinical phenotypes and thus develop treatment plans. Microarray analysis of monocytes with APS revealed 547 differentially expressed genes and identified novel miRNA-mRNA-intracellular signal regulatory networks in monocytes associated with CVD. In patients with APS, the monocytes showed reduced expression of miR-19b-3p and miR-20a-5p, and those with the least miRNA expression had the highest levels of aPL (190, 191). The results of the *in vitro* experiments revealed that APS patients had significantly higher serum levels of HMGB1 and sRAGE, as compared to healthy individuals. anti-β2-GPI antibody induced RAGE activation and HMGB1 cell relocalization in monocytes and platelets. As an increase in proinflammatory triggers, HMGB1/sRAGE may be involved in monitoring the risk of recurrent miscarriage (192). Monocytes and ECs have a pivotal role in the development of APS, and the interaction between monocytes and ECs has been described in the previous section. Ulštok's study was the first to show a marked increase in VLA4 on monocyte surfaces in patients with APS, and *in vitro* stimulation of Catastrophic APS (CAPS) showed a more significant increase in VLA4, suggesting that VLA4-enhanced monocyte adhesion is involved in thrombotic pathophysiology in APS (193) (Figure 3).

Co-culture of APS monocytes with LPS increased the expression of *IL-1β*, *IL-6*, *IL-23*, *TLR2*, *CCL2*, and *CXCL10* genes to improve the sensitivity to LPS and contribute to the formation of thrombosis, but the response was weak in healthy donor cells (194). Reduced levels of ATP result in decreased inflammation among APS monocytes by blocking unidentified signals stimulated by LPS and inhibiting ATP-mediated production of IL-1 through the inflammasome and IL-10 (194, 195). Immunomodulation of plasma exchange reversed the expression of IL-1 β, IL-6, IL-23, CCL2, P2X7, and TNF-α in APS monocytes, with mRNA expression levels returning to the normal range (196). For secondary thromboembolism in APS, it is generally accepted that direct oral anticoagulants can prevent this from occurring. There are currently many anticoagulant drugs of choice in clinical practice, such as DOAC rivaroxaban and warfarin drugs. Compared with warfarin, DOAC rivaroxaban not only provides anticoagulation but also reduces the frequency of bleeding events and does not require patient diet control (197).

### Atherosclerosis

Atherosclerosis is a slowly progressing condition of the large and medium-sized arteries, characterized by the gradual build-up of plaques over time. Thrombotic complications of atherosclerotic diseases, such as ACS, occur suddenly and often without warning signs. The long-term process of atherosclerotic plaque initiation and formation is thought to be accelerated by different risk factors, including traditional factors such as smoking, hypertension as mentioned above, hyperglycemia, and severe hyperlipidemia (198). It is increasingly believed that environmental factors, such as air pollution, noise, sleep disorders and stress, contribute to atherosclerotic events in part through the activation of inflammatory pathways (199). The inflammatory mechanisms run through the whole process of atherosclerosis formation, and mononuclear macrophages play a key role. In early atherosclerosis, LDL remains in the intima of blood vessels, mediated by oxidase, lipolysis, proteolytic enzymes, and ROS (200). A variety of danger-associated molecular patterns (DAMP) are modified to obtain immunogenicity. Immunogenic LDL activates vascular ECs and mobilizes immune cells (mainly monocytes and T cells) to inflammatory tissues, mediated by locally produced chemokines, as previously described in the context of arterial thrombosis. Chemokines (CCR2, CCR5, and CX3CR1) bind to receptors on monocytes to promote migration to tissues. When three chemokine receptors, CCR2, CCR5 and CX3CR1 were blocked, atherosclerosis in hypercholesterolemic mice was virtually eliminated by inhibiting the aggregation of monocytes into inflammatory tissue (201). This suggests that all monocyte subsets are involved in the formation of atherosclerosis.

The atherosclerotic process may be a training immune process (Figure 3). Studies have shown that monocytes, as innate immune cells, can form features of immune memory after brief exposure to microorganisms (202). Unlike the specific memory of adaptive immune cells, trained immunity is characterized by its nonspecificity and ability to induce a long-lasting proinflammatory phenotype in monocytes/macrophages through epigenetic reprogramming, particularly at the level of histone methylation (202). In addition, non-microbial endogenous stimuli can also induce trained immunity, and current studies include lipoproteins and adrenal hormones, hyperglycemia, and other factors that contribute to the development of atherosclerotic cardiovascular disease (ASCVD) (203, 204). Monocytes immunized by Ox-LDL training are induced through the Akt-mTOR-HIF-1α pathway, and intracellular metabolic glycolysis and oxidative phosphorylation are up-regulated, which is an important pathway to promote proinflammatory cytokines (205–207). Pharmacological inhibition of the mTOR pathway and related signaling molecules, as well as inhibition of 2-deoxyglucose glycolysis, prevents glycolysis, ROS formation, and proinflammatory initiation in monocytes/macrophages (206, 207). A recent study in 243 healthy volunteers showed that oxLDL-induced training immunity led to changes in the balance of steroid hormones within monocytes. Progesterone inhibits oxLDL-induced training immunity through ribocorticosteroid receptors and minerocorticoid receptors, an effect that may help reduce CVD risk in premenopausal women (208).

In addition to the promotion of cytokine proliferation, macrophages induced by Ox-LDL showed high expression of Ox-LDL recognition receptors CD36 and SR-A and low expression of anticholesterol receptors ABCAI and ABCG1, thus enabling macrophages to have a stronger lipid absorption capacity (209). These processes accelerate the formation of atherosclerosis. Differentiated macrophages, as key cells in atherosclerosis, are transformed into foam cells, and subsequent increase in cholesterol load over time causes the triggering of unfolded protein responses in the ER, from intracellular crystallization to precipitation and activation of inflammasomes, ultimately leading to programmed cell death (210). Foam cells accumulate to form an atherosclerotic core. The properties of lipid material, cholesterol crystallization, accumulation of foam cell fragments to form an atherosclerotic core, and ensuing thinning of the fibrous cap result in a high degree of thrombosis (211, 212). Interestingly, the investigators discovered that neovascularization was prevalent in atherosclerotic plaques, and highly neovascularized plaques were more susceptible to rupturing than their stable counterparts. Matrix metalloproteinases secreted by monocytes/macrophages were responsible for this phenomenon by deteriorating and redesigning the ECM and activating or disintegrating growth factors. As a result, stable atherosclerotic lesions may become unsteady high-risk plaques due to intraplaque hemorrhage.

Macrophages were thought to be major players in the formation of atherosclerosis as well as thrombotic complications. Although the connection between atherosclerosis and immunity in humans is constantly evolving, some of our understanding of this relationship comes from research conducted on animal models of CVD. However, due to inherent biological, genomic, and environmental differences, the results obtained therein cannot be fully transferable to the human environment. Potential therapeutic approaches against atherosclerosis include targeting monocytes/macrophages recruitment, polarization, cytokine profiling, ECM remodeling, cholesterol metabolism, oxidative stress, inflammatory activity, and non-coding RNA. Besides, folic acid can restore hyperlipidemia (HL) and hyperhomocysteinemia (HHcy)-mediated aberrant DNA methylation and decreased ARID5B expression, thereby inhibiting atherosclerotic plaque formation by reducing the proportion of intermediate monocytes (213).

In recent years, there has been significant interest in understanding the role of monocytes and monocyte-derived extracellular vesicles (MDEVs) in the major risk factors for atherosclerosis (214, 215). Several studies have reported a significant association between monocytes and obesity, a major risk factor for atherosclerosis (216, 217). These studies have shown that obesity leads to increased levels of chemokines and cytokines, promoting monocyte recruitment and activation. It also results in increased oxidative stress and inflammation, leading to MDEV release (218). MDEVs have been shown to exert pathogenic effects by promoting endothelial dysfunction and a pro-inflammatory state, exacerbating atherosclerosis (219). In addition, in a clinical trial of obese women without other cardiovascular risk factors, the researchers found that circulating procoagulant microparticles were increased in obese patients compared with healthy controls, and obese patients had an increased risk of thrombosis, suggesting that obesity as a major risk factor for atherosclerosis is associated with the release of MDEVs (220).

Diabetes is another major risk factor for atherosclerosis, with chronic hyperglycemia leading to increased oxidative stress and inflammation (221). High glucose levels induce monocyte activation, leading to MDEV release, and increased endothelial adhesion molecules, resulting in a pro-inflammatory and pro-thrombotic environment (222). The concentration of MDEVs in plasma of patients with type 2 diabetes was much higher than that of healthy individuals, and the amount of MDEVs was positively correlated with platelet activation markers (223, 224). Furthermore, MDEVs have been shown to induce endothelial dysfunction and increase monocyte and macrophage recruitment, aggravating atherosclerosis in diabetes (219).

Hypertension is another major risk factor for atherosclerosis, and monocytes are involved in its pathogenesis. A study showed that shear stress induces monocyte recruitment, activation and migration, promoting inflammation and neointimal formation (225). MDEVs have also been implicated in hypertension. In a clinical trial of 359 hypertensive patients, investigators observed increases in MDEVs and sVCAM-1 regardless of their presence or absence of diabetes (226). In another study, the expression of MDEVs and platelet activation markers (CD62P, CD63, PAC-1, and annexin V) was much higher in hypertensive patients than in healthy individuals, suggesting that hypertensive patients are more prone to atherosclerosis (227).

Hypercholesterolemia is also a cause of atherosclerosis. In response to high levels of cholesterol, monocytes can infiltrate the arterial wall and differentiate into macrophages, which can take up and accumulate lipids, leading to the formation of foam cells and ultimately the development of atherosclerotic plaques (228). Although the exact mechanism is not yet fully understood, recent studies suggest that MDEVs may also be involved in the development of atherosclerosis, and secretion of MDEVs by monocytes can activate endothelial cells and other immune cells and promote inflammation and further atherosclerotic plaque formation (229). Clinical trials have found that elevated oxidized LDL cholesterol in hypercholesterolemia induces increased release of MDEVs (230). MDEVs appear to be markers for the diagnosis of atherosclerotic plaques in patients with hypercholesterolemia, and the number of circulating microparticles before thrombosis can indicate atherosclerotic plaques in the subclinical stage of patients with hypercholesterolemia (231–233).

Other risk factors for atherosclerosis, such as smoking, aging, and dyslipidemia, have been associated with monocyte activation and MDEV release. Smoking induces oxidative stress and inflammation, leading to increased monocyte activation, and MDEV release (234). In cell experiments simulating smoking environments, tobacco smoke-exposed monocytes/macrophages have been found to induce biologically active procoagulant MDEVs in cells, which may be associated with activation of JNK, p38, ERK, and MAPK (235). Aging is a primary risk factor for atherosclerosis, characterized by declines in tissue and cell functions resulting in increased inflammation and oxidative stress, leading to MDEV release (236). Dyslipidemia, characterized by increased levels of LDL cholesterol, induces monocyte activation and lipid accumulation in macrophages, leading to foam cell formation and atherosclerosis (237). In addition, IL-33 acts as a pro-inflammatory factor, induces TF expression in monocytes, releases procoagulant MDEVs, and ultimately forms a prothrombotic state of atherosclerosis (238).

In summary, monocytes and monocyte-derived extracellular vesicles play a crucial role in the pathogenesis of atherosclerosis, and different major risk factors for the disease have been associated with their activation and release (239). Obesity, diabetes, hypertension, smoking, aging, and dyslipidemia all promote monocyte activation and MDEV release, leading to increased inflammation, oxidative stress, and endothelial dysfunction, culminating in atherosclerosis. A better understanding of the role of monocytes and MDEVs in atherosclerosis could lead to novel therapeutic interventions targeting their recruitment and activation, potentially preventing or slowing the progression of this debilitating disease.

### Rheumatic heart disease (RHD)

RHD is a condition caused by damage to the heart valves from rheumatic fever (RF), which is an autoimmune response to untreated streptococcal throat infections (240). Left atrial (LA) thrombosis is a common complication with RHD and mitral stenosis (MS) (241). As early as 1991, the luminol-enhanced chemiluminescence technique was used to study the production of oxygen free radicals (OFR) by monocytes and neutrophils in the peripheral blood of patients with RF and RHD (242). It has been observed that in RHD patients, phagocytes are capable of penetrating the myocardium and possibly contributing to the development of cardiac injury by generating OFR (242). In a clinical study of mitral valve resection in patients with rheumatic MS, histological studies have revealed a positive correlation between the degree of infiltration of inflammatory cells in valve tissue and the levels of MMP-1 and IFN-γ. In addition, in an *in vitro* cell assay, IFN-γ was found to increase MMP-1 expression in monocytes (243). The risk of embolus dislodging and causing thromboembolism may increase due to the easy tearing of valve tissue resulting from the thickening of collagen and structural abnormalities in the valve tissue caused by inflammatory cell infiltration.

The expression of inflammatory cells and inflammatory genes in the aortic valve of female RHD patients was significantly higher than that of male patients. More monocytes and macrophages infiltrate into the aortic valve to produce IFNγ and IL8, which as major Th1 cytokines, participate in the positive feedback between macrophages and Th1 cells to accelerate the secretion of pro-inflammatory cytokines. The NFKB pathway plays a crucial role in enhancing the autoimmune response of the aortic valve in female RHD patients by increasing mononuclear/macrophage infiltration and pro-inflammatory cytokine (especially Th1 cytokine) levels. Upon activation, phosphorylated p65 enters the nucleus and triggers the transcription of inflammatory genes, which further exacerbates the inflammation of the damaged aortic valve (244).

RF is a complex inflammatory autoimmune disease that involves the interaction of multiple pro-inflammatory agents produced by activated neutrophils and macrophages, resulting in a synergistic pathological effect (245). In individuals with rheumatic mitral stenosis and atrial fibrillation, pro-inflammatory M1 macrophages are primarily observed in the atria of those with mitral stenosis, atrial fibrillation, and thrombosis. The inflammatory effect of M1 cells may be related to the up-regulation of eNOS expression. In addition, the polarization of M1 macrophages towards M2 is inhibited due to the down-regulation of M-CSF (246).

### Deep venous thrombosis (DVT)

DVT is a kind of thrombotic disease. It refers to venous reflux disorders caused by abnormal coagulation of blood in deep veins (247). It often occurs in the lower limbs. It is a common and serious complication of hospitalized patients, especially those after major surgery and long-term bedridden patients, with high incidence and serious consequences. Detachment of the thrombus can cause PE, and both are collectively referred to as VTE.

The mononuclear/macrophage system is essential in both the formation and resolution of DVT. In mice with venous thrombosis, inflammatory bodies are activated, triggering venous thrombosis through pyrodeath and TFs released by inflammatory monocytes and macrophages (248). Lack of caspase-1 but not caspase-11 protects mice from venous thrombosis. The monocyte signaling pathway, key enzymes and thrombolytic regulators regulate the occurrence of DVT, which can be used as a molecular target network to study the treatment of DVT. Protein tyrosine kinases participate in the activation of monocytes and ECs. For example, tyrosine kinase PYK2 regulates platelet activity and TF expression by stimulating monocytes and ECs, and is involved in the regulation of natural immunity and inflammation (249). In a mouse experiment, the number of inflammatory Ly6^Chi^ monocytes controls DVT formation, growth, and regression, and Nur77 agonist may be an ideal candidate for therapeutic intervention with inflammatory monocyte activity in patients with DVT to prevent thrombosis growth and accelerate regression (250). Inhibit the formation of Tbet^+^ interleukin-12 from bone marrow monocytes and accelerate thrombus regression (251). Modulating inflammatory immune responses may be a way to improve DVT treatment. The effects of MMP-9 on the loss of compliance during thrombolysis and the biomechanics of vein walls contribute to the development of specific molecular therapies for DVT. With the age of thrombosis, IFN-γ is mainly infiltrated by macrophages, and IFN-γ/STAT1 signaling pathway is activated to negatively regulate the expression of *Mmp-9* and *Vegf* genes in PMA-induced macrophages (252). The use of anti-IFN-γ monoclonal antibody can accelerate the resolution of DVT. Recombinant human granulocyte colony-stimulating factor (rhG-CSF) not only mobilized monocyte lineage cells into peripheral blood but also induced higher expression of CCR2 protein, thus enhancing the regression and recalculation of venous thrombosis (253). Circulating monocytes and monocyte-derived macrophages in patients with idiopathic DVT exhibit significantly increased M1 polarization, which can significantly up-regulate the expression of endothelial cell adhesion molecules (134). The thrombus targeting adenovirus uPA (ad-uPA) gene transduction of human blood monocyte-derived macrophages (HBMMs) increases their fibrinolytic activity. In an experimental model of venous thrombosis, systematic administration of uPA up-regulated HBMMs reduced the size of the thrombus (254). Alternatives to delivering fibrinolytic agents are worth exploring. Loss of prostag-landin-endoperoxide synthase (PTGS) alters the natural distribution of ANXA2 in mononuclear/macrophages, increases TF expression and activity, and leads to venous thrombosis. Targeting PTGI2/ANX2/TF pathways, such as treatment with cabpaprost, inhibits nuclear ANXA2 transport, controls monocyte TF activity, and prevents the occurrence of DVT (255).

### Diabetic nephropathy (DN)

DN has emerged as a pervasive renal disease, causing end-stage renal failure on a global scale (256). As one of the most serious and harmful chronic complications caused by diabetes, DN is one of the manifestations of diabetic systemic microangiopathy (257). DN has a prethrombotic state and obvious endothelial dysfunction (258). With the increase of urinary protein and the progression of the disease, it is easy to appear hypercoagulation, and then thrombosis.

Inflammatory cell infiltration is a consistent feature of the early stages of DN, particularly the influx of mononuclear cells into the affected tissues and the infiltration of macrophages into the kidney (259, 260). This influx is thought to be due to the activation of innate immunity accompanied by the development of a chronic low-grade inflammatory response, but may also be related to differences in monocyte phenotype and function. Experiments showed that diabetes increased the number of monocytes and had no effect on the total number of white blood cells. At the same time, the number of CD14^+^CD16^++^ NCMs was significantly reduced by diabetic status, but only in patients with diabetic complications (261). The circulating monocytes phenotype can be changed by diabetic complication status. Changes in monocyte activation and function have also been reported in inflammatory pathways in diabetes, such as increased monocyte adhesion to ECs and enhanced TLR signaling pathway conduction in monocytes (262). Mouse data showed that monocyte CD163 and sCD163 changes predate DN. CD163+ monocytes with a high circulating proportion have anti-inflammatory effects and may protect against diabetes complications (263).

Macrophage infiltration and activation are evident in the renal biopsies of diabetic animal models and patients with DN (264–266). Therefore, monocytes need to be activated and migrate from the circulation to differentiate and infiltrate the renal mesangium. Under the influence of high glucose, monocyte adhesion, migration, differentiation and MMP expression can be enhanced by increasing the secretion of pro-inflammatory cytokines in mesangial cells, and thus MMPs can mediate the degradation of ECM (260, 267). Mesangial cell-monocyte interactions are important to activate monocyte migration from circulation to the kidney in early DN. The pro-inflammatory cytokines TNF-α and IL-6 can induce the expression and adhesion of MMP-9 in monocytes (260). Moreover, the interaction between monocytes and human renal mesangial cells (HRMCs) is also through MMP-2 and fractalkine. In the Monocyte-HRMC co-culture system, AGEs not only directly down-regulated MMP-2, but also indirectly down-regulated the expression and activity of MMP-2 through up-regulated fractalkine, thus increasing the expression of fractalkine, which is mainly used as chemotactic agent (268) (Figure 3). The presence of such crosstalk between monocytes and HRMCs has been interpreted to be related to abnormal deposition of ECM and migration of monocytes into tissues.

Diabetes can induce renal MCP-1 production, and serum and urinary MCP1 levels are elevated in early DN, which can be used as markers and possible mediators of early DN, as well as to evaluate renal inflammation in diabetic patients (269, 270). Increased expression of MCP-1/CCL2 in the kidney is considered a crucial contributor to the initiation of monocyte recruitment in the tubulointerstitium (271, 272). Exposure to MCP-1 and high-glucose environments induces infiltration and activation of macrophages, resulting in the release of ROS, profibrotic growth factor (TGF-β, PDGF) and pro-inflammatory cytokines (IL-1, TNF-α, MCP-1) (272–274). The amplified inflammatory response mediates parenchymal cell injury and death leading to decreased renal function, and myofibroblast proliferation promotes renal fibrosis. These tandem processes together promote the progression of DN, lead to the development of kidney failure and cause vascular dysfunction. Inhibition of MCP-1 in DN shows strong therapeutic potential by reducing persistent proteinuria. Spiegelmer® emapticap pegol (NOX-E36) continued to restore glomerular endothelial glycocalyx and barrier function, decreased CCR2-expressing Ly6^Chi^ monocytes in peripheral blood, and polarization of tissue macrophages toward anti-inflammatory phenotypes (275). Activation of the renin-angiotensin system (RAS) is a characteristic manifestation of DN (276).

Activation of RAS is pro-inflammatory, and pro-fibrotic and enhances oxidative stress. By increasing the production of MCP-1 and osteopontin in renal tubule cells, it facilitates the adhesion of monocytes to renal ECs and thus their entry into the kidney. Under the activation of various proinflammatory cytokines and RAS, macrophages differentiate into M1 proinflammatory phenotypes, leading to the development of DN. Under the antagonistic drugs of RAS, the phenotypic transformation of macrophages and the number of infiltrates can be improved, thus reducing proteinuria and correcting renal disorders (277, 278). Furthermore, recent studies have shown that signal communication between macrophages and renal tubular epithelial cells (TECs) can be used as targets in the treatment of DN. For example, the exosome/miR19b-3p/SOCS1 axis mediates the communication between injured TECs and macrophages, leading to the activation of M1 macrophages (279). During DN progression, IRG1 enrichment, lipotoxic tec derived EVe activates macrophages through TGFβR1-dependent pathways and subsequently up-regulates the expression of many inflammatory genes, thereby inducing inflammation and damage in DN. Meanwhile, the LRG1/TGFβR1 signaling pathway can also increase the expression of TRAIL in macrophages, and the macrophage-derived EVs enriched with TRAIL can promote the apoptosis of TECs, acting as a feedback loop in DN (280). Proteinuria induces glycolysis in renal macrophages by stabilizing HIF-1α through tubular epithelial-derived EVs (281). These targets may be some important links to subsequent regulation, which needs further in-depth study.

Indicators related to monocytes can be an important predictor of DN, and increased Monocyte-lymphocyte ratio (MLR) is significantly associated with the risk of DN (282). Higher monocyte to high-density lipoprotein cholesterol ratio (MHR) levels for patients with DN compared to others (283).

## Conclusion

Monocytes undoubtedly have a significant role in the pathophysiology of various thrombotic diseases. Compared with tissue macrophages, circulating monocytes are easier to detect and characterize. As the medical field advances into the 21st century, with an augmented emphasis on forecasting and averting future acute phenomena, the enumeration and functionality of monocytes and their respective subsets have the potential to serve as an alluring biomarker. Nevertheless, crucial parameters of monocytes that have the capability to act as prognostic indicators and targeted therapies necessitate identification and authentication.

While monocytes have been implicated in the link between inflammation and the pro-thrombotic state, their potential use as disease biomarkers or therapeutic agents in thrombosis is still an area of ongoing research. While there have been some promising findings, there are also challenges associated with the use of monocytes in these contexts. For example, monocyte populations are heterogeneous, and their pro-thrombotic or anti-thrombotic properties may vary depending on their differentiation state and activation status. Additionally, the interaction between monocytes and other immune cells, such as platelets and neutrophils, can further complicate the picture. Therefore, while the role of monocytes in thrombotic disease is an important area of investigation, it may be premature to consider them as biomarkers or therapeutic agents without further research and clinical validation. Further studies on the specific molecular mechanisms underlying the association between monocyte activation and thrombosis could inform the potential development of new targeted interventions for thrombotic disease.

In these thrombotic diseases, we found that each disease can also be closely related to each other, and monocytes act as an indispensable part as a close link in this network. Clarifying the role of monocytes in thrombotic diseases is helpful to prevent and diagnose thrombotic diseases, to reduce the incidence and mortality of thrombotic diseases.
